# Statistical CSI-Based Design for Pinching Antenna Systems with Short-Packet Communication

**DOI:** 10.3390/e28070722

**Published:** 2026-06-24

**Authors:** Zian Pan, Guansan Zheng, Zixuan Xu, Lei Yuan

**Affiliations:** 1 School of Materials & Energy, Lanzhou University, Lanzhou 730000, China; panza2023@lzu.edu.cn; 2 School of Information Science and Engineering, Lanzhou University, Lanzhou 730000, China; zhenggs2024@lzu.edu.cn (G.Z.); xuzx2023@lzu.edu.cn (Z.X.)

**Keywords:** pinching antenna, deep reinforcement learning (DRL), statistical channel state information (CSI), short-packet communication, ultra-reliable and low-latency communication (URLLC)

## Abstract

This paper designs a statistical channel state information-based pinching antenna system for short-packet communication (SPC). To maximize the average maximal achievable rate (MAR) under physical collision-avoidance constraints, we formulate a highly non-convex geometry optimization problem, which is solved by our proposed novel phase-domain proximal policy optimization (PPO) framework. Unlike conventional coordinate-based approaches, the agent operates in a dual-component trigonometric phase domain, and the generated phase actions are mapped to feasible antenna positions via a customized phase-domain action mapping, which fundamentally avoids the 0/2π phase discontinuity and ensures stable learning. To evaluate the reliability of SPC, we derive a tractable statistical characterization of the received signal-to-noise ratio based on a mixture Gamma approximation over spatially correlated Rician fading channels, leading to a closed-form approximation for the average block error rate (BLER). A bisection search algorithm is further developed to minimize the required blocklength under the target reliability constraint. Simulation results demonstrate that the proposed phase-domain PPO scheme significantly outperforms the conventional algorithms in terms of average MAR, average BLER, and blocklength efficiency, with the performance gain becoming more pronounced as the number of antennas per waveguide increases.

## 1. Introduction

Sixth-generation (6G) wireless networks are envisioned to provide unprecedented connectivity, particularly demanding ultra-reliable and low-latency communication (URLLC) [1]. To meet the stringent latency and reliability requirements of URLLC, communication in high-frequency bands (e.g., millimeter-wave and terahertz) has been widely recognized as a key enabler due to their abundant spectral resources [2]. However, high-frequency signals suffer from severe free-space path loss and are highly susceptible to blockages. To overcome this issue, reconfigurable holographic surface (RHS)-based large-scale antennas and pinching antennas (PAs) have recently attracted significant attention as novel flexible antenna architectures. Unlike RHS-based antennas, which use metasurface design to replace phase shifters [3], pinching antenna systems (PASSs) achieve similar flexibility through mechanical position tuning [4]. Specifically, in PASSs, pinching antennas (PAs) are mechanically coupled onto dielectric waveguides and can dynamically adjust their radiation positions by continuously sliding along the waveguides. Compared with conventional antenna types such as Yagi-Uda, patch antennas, and fixed arrays, PASSs significantly reduce hardware complexity and power consumption while fully exploiting the continuous spatial degrees of freedom (DoFs) [5,6].

To date, PASSs have been extensively studied in the context of conventional long-packet communications, where classic Shannon capacity serves as the fundamental performance metric [7,8,9,10,11]. For instance, the system performance of PASSs has been characterized in terms of outage probability and ergodic capacity in [7], and its application in physical-layer security has been explored in [8]. However, these studies operate under the infinite-blocklength assumption, which is inadequate for URLLC applications such as factory automation [12], where sensor/actuator data ranging from tens to hundreds of bits must be delivered with sub-millisecond latency and ultra-high reliability. In contrast, short-packet communication (SPC) is a key technology in realizing URLLC in the finite-blocklength regime, accurately characterizing the performance boundary under low-latency constraints [13]. The maximal achievable rate (MAR) in SPC is penalized by channel dispersion and the desired block error rate (BLER) [14]. SPC has been successfully integrated into various wireless communication systems [15,16,17,18,19]. However, the integration of SPC into PASS networks is still in its infancy. The work in [20] conducted the first analysis of the BLER performance of SPC for PASSs and verified the significant advantages of PASSs over conventional fixed-antenna systems. This demonstrates that the fusion of PASSs and SPC offers a promising pathway for URLLC.

However, the work in [20] is confined to deterministic channel models without incorporating the randomness of small-scale fading. While such deterministic models may provide a performance upper bound, they are inadequate for URLLC scenarios where even rare fading events can cause decoding errors due to the channel dispersion effect in the finite-blocklength regime. Furthermore, obtaining instantaneous channel state information (CSI) over fading channels, which incurs substantial channel estimation overhead, is practically challenging in URLLC scenarios due to stringent latency constraints. In contrast, statistical CSI can be acquired with much lower overhead [21,22,23]. Thus, PASS design based on statistical CSI over fading channels is more suitable for URLLC applications because of its reduced estimation overhead and implementation complexity. Additionally, in densely deployed PASS scenarios where multiple PAs slide along the same waveguide, the spatial channels exhibit severe correlation. The effective signal-to-noise ratio (SNR) is determined not only by the phase shifts derived from physical positions but also by this spatially correlated fading, posing a significant challenge for the theoretical characterization of the BLER. More critically, optimizing the performance of SPC for PASSs requires continuous optimization of the antenna geometry (i.e., the physical positions of PAs) while strictly satisfying physical collision avoidance constraints on the waveguides, which inherently formulates a highly non-convex optimization problem. Traditional heuristic algorithms, such as particle swarm optimization (PSO) [24], suffer from slow convergence and are prone to local optima when searching in high-dimensional continuous spaces. Deep reinforcement learning (DRL) has shown great potential in solving complex resource allocation problems [25,26,27]. For example, a deep reinforcement learning framework is proposed in [28] to solve redundant transmission scheduling over Gilbert–Elliot channels for remote state estimation, ensuring system stability under unreliable wireless links. A prior-guided goal-conditioned twin delayed deep deterministic policy gradient (TD3) algorithm is developed in [29] to automate antenna design, combining supervised learning with reinforcement learning to efficiently optimize antenna structural parameters. However, directly applying standard DRL algorithms, e.g., proximal policy optimization (PPO) [25] and TD3 [27], to optimize continuous antenna positions often struggles in convergence. This limitation fundamentally arises because the received signal quality is governed by phase alignment. Due to the periodic nature of electromagnetic waves, multiple distinct physical positions can yield the exact same effective phase, creating a highly irregular and degenerate reward landscape. Consequently, the agent struggles with this many-to-one mapping and fails to capture the underlying physics of coherent signal combination.

To address these challenges, this paper designs a statistical CSI-based PASS for SPC over spatially correlated Rician fading channels [30]. Specifically, we propose a novel phase domain PPO framework to optimize the system design. By executing policy learning directly in the phase domain and then mapping the learned actions back to the physical position domain, we achieve geometry optimization for user locations. The main contributions of this paper are summarized as follows.

- First, we design a statistical CSI-based PASS for SPC over spatially correlated Rician fading channels. We formulate an antenna geometry optimization problem to maximize the average MAR under blocklength and BLER constraints, while satisfying physical collision-avoidance constraints among moving antennas on parallel waveguides. To solve the resulting highly non-convex optimization problem, we propose a phase-domain PPO framework. Specifically, the agent exploits the phase structure of the received signal and operates in a dual-component trigonometric phase domain. The resulting actions are then mapped to feasible antenna positions via a customized phase-domain action mapping (PDAM). This design improves learning efficiency while ensuring collision-free deployment.
- Second, to analytically characterize the URLLC performance, we derive an approximate closed-form expression for the average BLER based on the derivation of the cumulative distribution function (CDF) of the received SNR. Based on this result, we propose a bisection search algorithm to minimize the blocklength under the user’s average BLER constraint.
- Finally, simulation results validate the accuracy of our analytical derivations. Furthermore, the proposed phase-domain PPO algorithm outperforms the existing baselines, including PSO [24], PPO [25], soft actor-critic (SAC) [26], and TD3 [27], in terms of average MAR and average BLER. This performance gain becomes more pronounced as the number of PAs per waveguide increases.

Notations: Table 1 summarizes the key symbols used in this paper.

## 2. System Model and Problem Formulation

**Figure 1:**
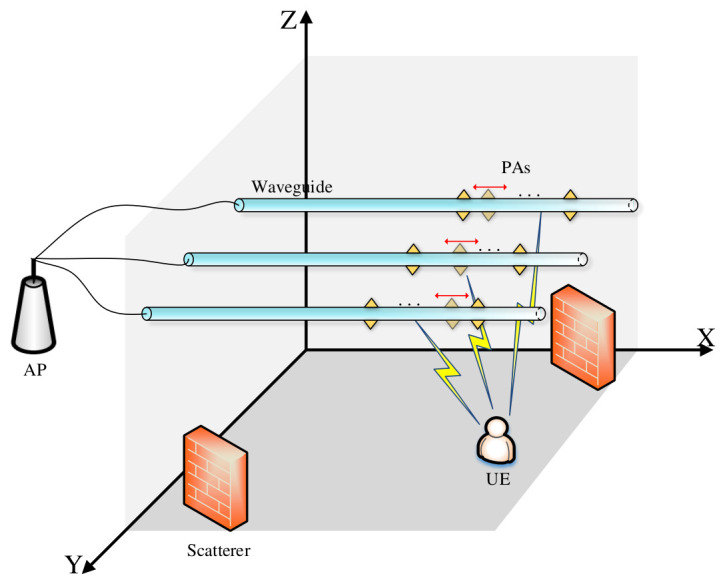
Illustration of our considered system.

As shown in Figure 1, we consider a downlink SPC system aided by a PASS operating in a high-frequency band. The Access Point (AP) is equipped with *N* parallel dielectric waveguides, each driven by an independent radio frequency chain. To enable dynamic beamforming, *M* PAs are mechanically coupled onto each waveguide. A single-antenna user equipment (UE) is located at a random position $p_{u}={\left[x_{u},y_{u},0\right]}^{T}$ within the service coverage area. Let N={1,…,N} and M={1,…,M} denote the sets of waveguides and antennas, respectively. The position of the *m*-th antenna on the *n*-th waveguide is denoted by the coordinate $x_{n,m}\in \left[0,L_{\max}\right]$, where $L_{\max}$ denotes the maximum length of the waveguide. The antenna array geometry vector $x_{n}={\left[x_{n,1},\ldots ,x_{n,M}\right]}^{T}$ must satisfy the physical collision avoidance constraint, i.e.,

(1) $$x_{n,m}-x_{n,m-1}\geq \Delta x,\;\forall n\in N,\;m=2,\ldots ,M,$$

where Δx is the minimum required spacing (e.g., the physical width of the antenna fixture). Consequently, the full antenna geometry matrix is defined as $X={\left[x_{1},\ldots ,x_{N}\right]}^{T}\in R^{N\times M}$. Notably, in our considered URLLC scenario, the UE is considered to be quasi-stationary during the short-packet transmission. The mechanical movement of PAs and short-packet transmission are decoupled in time. Specifically, antennas are repositioned slowly and then remain stationary during low-latency transmission. Additionally, to reduce channel estimation overhead, we adopt statistical CSI for beamforming. For simplicity, in this paper we assume that the AP has perfect knowledge of both the statistical CSI and the UE’s location. The impact of channel estimation errors, localization uncertainty, and outdated CSI on system performance will be analyzed in our future work. This work acts as a first step to establish the theoretical performance upper bound of PASS-aided SPC systems using statistical CSI.

### 2.1. Waveguide Signal Transmission

The signal propagation within the PASS consists of in-waveguide transmission and radiation coupling. According to coupled mode theory [32], the signal radiates from the PAs as it propagates along the waveguide. Let s∈C be the transmitted symbol satisfying $E[|s{|}^{2}]=1$. The signal radiated from the *m*-th antenna on the *n*-th waveguide, denoted by $u_{n,m}$, is given by

(2) $$u_{n,m}=\sqrt{P_{B}}\alpha_{m}w_{n}e^{-j\beta_{g}x_{n,m}},$$

where $P_{B}$ is the total transmit power, $\alpha_{m}$ is the radiation coefficient, $w_{n}$ is the digital beamforming coefficient for the *n*-th waveguide with $\sum_{n=1}^{N}{\left|w_{n}\right|}^{2}=1$, $\beta_{g}=2\pi /\lambda_{g}$ is the propagation constant within the waveguide, and $\lambda_{g}$ is the wavelength in the waveguide. To ensure practical feasibility, we adopt the proportional power model proposed in [33], where $\alpha_{m}$ is designed to be

(3) $$\alpha_{m}=\delta {\left(\sqrt{1-\delta^{2}}\right)}^{m-1},$$

and δ is the coupling factor determined by the antenna structure.

### 2.2. Spatially Correlated Rician Channel Model

We consider a realistic spatially correlated Rician fading channel. The channel coefficient between the *m*-th antenna on the *n*-th waveguide and the UE is modeled as

(4) $$h_{n,m}=\sqrt{\beta_{n,m}}\left(\sqrt{\frac{K}{K+1}}{\bar{h}}_{n,m}+\sqrt{\frac{1}{K+1}}h_{n,m}^{\mathrm{NLoS}}\right),$$

where *K* is the Rician factor, and $\beta_{n,m}=\xi_{0}{∥p_{n,m}-p_{u}∥}^{-\eta }$ represents the large-scale path loss with reference loss $\xi_{0}$ and path loss exponent η. Here, $p_{n,m}={\left[x_{n,m},\left(n-1\right)d_{wg},H\right]}^{T}$ is the three-dimensional position of the *m*-th antenna on the *n*-th waveguide with $d_{wg}$ and H denoting the adjacent waveguide spacing and the deployment height, respectively. The line-of-sight (LoS) component in $h_{n,m}$ is determined by the geometric distance, i.e.,

(5) $${\bar{h}}_{n,m}=e^{-j\frac{2\pi }{\lambda }∥p_{n,m}-p_{u}∥},$$

where λ is the carrier wavelength in free space. Due to the dense deployment of PAs on the waveguide, the none-LoS (NLoS) components exhibit strong spatial correlation. We define the NLoS channel vector for the *n*-th waveguide as $h_{n}^{\mathrm{NLoS}}={\left[h_{n,1}^{\mathrm{NLoS}},\ldots ,h_{n,M}^{\mathrm{NLoS}}\right]}^{T}$. To accurately model this correlation, we introduce the spatial correlation matrix $R_{n}\in C^{M\times M}$. The relationship between the correlated channel vector $h_{n}^{\mathrm{NLoS}}$ and the independent scattering components is given by [34]

(6) $$h_{n}^{\mathrm{NLoS}}=C_{n}{\tilde{h}}_{n},$$

where ${\tilde{h}}_{n}∼\mathrm{CN}\left(0,I_{M}\right)$ is a vector of independent and identically distributed (i.i.d) complex Gaussian variables, representing the rich scattering environment. $C_{n}$ is the Cholesky decomposition factor of the correlation matrix $R_{n}$, satisfying $R_{n}=C_{n}C_{n}^{H}$. Under the assumption of isotropic scattering, the entries of the spatial correlation matrix $R_{n}$ are determined by the antenna spacing through the sinc function [34]:

(7) $${\left[R_{n}\right]}_{i,j}=\mathrm{sinc}\left(\frac{2|x_{n,i}-x_{n,j}|}{\lambda }\right),\;i,j\in M.$$

It is worth noting that while antennas on the same waveguide are correlated, the spacing between different parallel waveguides is sufficient to assume statistical independence between waveguides. Let us define $R_{\mathrm{sys}}=\mathrm{diag}\left(R_{1},\ldots ,R_{N}\right)$. Thus, according to the superposition principle, the received signal *y* at the UE is the sum of the signals from all *N* waveguides and *M* antennas, i.e.,

(8) $$y=\sqrt{P_{B}}h_{\mathrm{air}}^{H}Gws+n_{0},$$

where $h_{\mathrm{air}}={\left[h_{1}^{T},\ldots ,h_{N}^{T}\right]}^{T}\in C^{NM\times 1}$, $h_{n}={\left[h_{n,1},\ldots ,h_{n,M}\right]}^{T}\in C^{M\times 1}$, and $w={\left[w_{1},\ldots ,w_{N}\right]}^{T}\in C^{N\times 1}$. Here, $n_{0}∼\mathrm{CN}\left(0,\sigma^{2}\right)$ represents the additive white Gaussian noise (AWGN). Specifically, the AP adopts a statistical CSI-based maximum ratio transmission (MRT) scheme, where the active beamforming vector is given by $w=\frac{G^{H}{\bar{h}}_{\mathrm{air}}}{∥G^{H}{\bar{h}}_{\mathrm{air}}∥}$ [35] with ${\bar{h}}_{\mathrm{air}}={\left[{\bar{h}}_{1}^{T},\ldots ,{\bar{h}}_{N}^{T}\right]}^{T}\in C^{NM\times 1}$ and ${\bar{h}}_{n}={\left[{\bar{h}}_{n,1},\ldots ,{\bar{h}}_{n,M}\right]}^{T}\in C^{M\times 1}$.

The overall in-waveguide channel matrix is given by

(9) $$G=\left[\begin{array}{cccc} g(x_{1}) & 0 & \cdots & 0\\ 0 & g(x_{2}) & \cdots & 0\\ \vdots & \vdots & \ddots & \vdots \\ 0 & 0 & \cdots & g(x_{N}) \end{array}\right]\in C^{NM\times N},$$

where $g\left(x_{n}\right)\in C^{M\times 1}$ represents the propagation vector within the *n*-th waveguide, i.e.,

(10) $$g\left(x_{n}\right)={\left[\alpha_{1}e^{-j\beta_{g}x_{n,1}},\ldots ,\alpha_{M}e^{-j\beta_{g}x_{n,M}}\right]}^{T}.$$

Defining the overall channel coefficient as $h_{\mathrm{total}}≜h_{\mathrm{air}}^{H}Gw$, we have the instantaneous received SNR

(11) $$\gamma =\rho |h_{\mathrm{total}}{|}^{2}=\rho {\left|\sum_{n=1}^{N}\sum_{m=1}^{M}\alpha_{m}e^{-j\beta_{g}x_{n,m}}w_{n}h_{n,m}\right|}^{2},$$

where $\rho =P_{B}/\sigma^{2}$ is the transmit SNR and $w_{n}$ is

(12) $$w_{n}=\frac{g{\left(x_{n}\right)}^{H}{\bar{h}}_{n}}{∥G^{H}{\bar{h}}_{\mathrm{air}}∥}=\frac{\sum_{l=1}^{M}\alpha_{l}{\bar{h}}_{n,l}^{*}e^{j\beta_{g}x_{n,l}}}{\sqrt{\sum_{k=1}^{N}{\left|\sum_{l=1}^{M}\alpha_{l}e^{j\left(\beta_{g}x_{k,l}+\frac{2\pi }{\lambda }∥p_{k,l}-p_{u}∥\right)}\right|}^{2}}}.$$

## 3. Statistical Analysis and Optimization

In this section, we first analyze the statistical properties of γ and then formulate the antenna geometry optimization problem. Since direct position optimization via DRL often suffers from oscillation due to the high phase sensitivity, we propose a novel phase-domain PPO algorithm to achieve robust and fast convergence.

### 3.1. Statistical Characterization and Problem Formulation

To derive the probability distribution of γ and facilitate performance analysis, we first express $h_{\mathrm{total}}$ as

(13) $$\begin{array}{c} h_{\mathrm{total}}=A_{1}+A_{2}, \end{array}$$

where

(14) $$\begin{array}{cc} A_{1} & ≜\sqrt{\frac{K}{K+1}}\sum_{n=1}^{N}\sum_{m=1}^{M}\alpha_{m}e^{-j\beta_{g}x_{n,m}}w_{n}\sqrt{\beta_{n,m}}{\bar{h}}_{n,m}\\ \; & =\frac{\sqrt{\frac{K}{K+1}}\sum_{n=1}^{N}\sum_{m=1}^{M}\alpha_{m}e^{-j\beta_{g}x_{n,m}}\sqrt{\beta_{n,m}}{\bar{h}}_{n,m}\sum_{l=1}^{M}\alpha_{l}{\bar{h}}_{n,l}^{*}e^{j\beta_{g}x_{n,l}}}{\sqrt{\sum_{k=1}^{N}{\left|\sum_{l=1}^{M}\alpha_{l}e^{j\left(\beta_{g}x_{k,l}+\frac{2\pi }{\lambda }∥p_{k,l}-p_{u}∥\right)}\right|}^{2}}} \end{array}$$

and

(15) $$\begin{array}{cc} A_{2} & ≜\sqrt{\frac{1}{K+1}}\sum_{n=1}^{N}\sum_{m=1}^{M}\alpha_{m}e^{-j\beta_{g}x_{n,m}}w_{n}\sqrt{\beta_{n,m}}h_{n,m}^{\mathrm{NLoS}}\\ \; & =\frac{\sqrt{\frac{1}{K+1}}\sum_{n=1}^{N}\sum_{m=1}^{M}\alpha_{m}e^{-j\beta_{g}x_{n,m}}\sqrt{\beta_{n,m}}h_{n,m}^{\mathrm{NLoS}}\sum_{l=1}^{M}\alpha_{l}{\bar{h}}_{n,l}^{*}e^{j\beta_{g}x_{n,l}}}{\sqrt{\sum_{k=1}^{N}{\left|\sum_{l=1}^{M}\alpha_{l}e^{j\left(\beta_{g}x_{k,l}+\frac{2\pi }{\lambda }∥p_{k,l}-p_{u}∥\right)}\right|}^{2}}}. \end{array}$$

Here, $A_{1}$ is a deterministic term, and $A_{2}$ follows $\mathrm{CN}(0,\sigma_{A_{2}}^{2})$ with

(16) $$\begin{array}{cc} \sigma_{A_{2}}^{2} & =\frac{1}{K+1}\sum_{n=1}^{N}{\left|w_{n}\right|}^{2}\sum_{i=1}^{M}\sum_{j=1}^{M}{\left[R_{n}\right]}_{i,j}\sqrt{\xi_{0}d_{n,i}^{-\eta }}\alpha_{i}\sqrt{\xi_{0}d_{n,j}^{-\eta }}\alpha_{j}e^{j\beta_{g}\left(x_{n,i}-x_{n,j}\right)}\\ \; & =\frac{1}{K+1}{\left(\sum_{k=1}^{N}{\left|\sum_{l=1}^{M}\alpha_{l}e^{j(\beta_{g}x_{k,l}+\frac{2\pi }{\lambda }∥p_{k,l}-p_{u}∥)}\right|}^{2}\right)}^{-1}\sum_{n=1}^{N}{\left|\sum_{m=1}^{M}\alpha_{m}e^{j(\beta_{g}x_{n,m}+\frac{2\pi }{\lambda }∥p_{n,m}-p_{u}∥)}\right|}^{2}\\ \; & \;\times \left[\sum_{i=1}^{M}\alpha_{i}^{2}\beta_{n,i}+2\sum_{i=1}^{M-1}\sum_{j=i+1}^{M}\mathrm{sinc}\left(\frac{2|x_{n,i}-x_{n,j}|}{\lambda }\right)\sqrt{\beta_{n,i}\beta_{n,j}}\alpha_{i}\alpha_{j}\cos\left(\beta_{g}\left(x_{n,i}-x_{n,j}\right)\right)\right]. \end{array}$$

and $d_{n,i}≜∥p_{n,i}-p_{u}∥$. Thus, we have $h_{\mathrm{total}}∼\mathrm{CN}\left(\mu_{h},\sigma_{h}^{2}\right)$ with $\mu_{h}=A_{1}$ and $\sigma_{h}^{2}=\sigma_{A_{2}}^{2}$.

For the given blocklength L (with L>100), BLER ε, and instantaneous SNR γ, the MAR in bits per channel use (BPCU) is approximated as [14]

(17) $$R\left(\gamma \right)\approx \log_{2}\left(1+\gamma \right)-\sqrt{\frac{V(\gamma )}{L}}Q^{-1}\left(\varepsilon \right),$$

where $V\left(\gamma \right)=\left(1-{\left(1+\gamma \right)}^{-2}\right){\left(\log_{2}e\right)}^{2}$ is the channel dispersion, and $Q^{-1}\left(\cdot \right)$ is the inverse Gaussian Q-function. Furthermore, for the given L and ε, the average MAR $\bar{R}=E\left[R\left(\gamma \right)\right]$ is an important performance metric in SPC over fading channels. According to [36] (Equation (8)), in the high-SNR regime, $\bar{R}$ can be approximated as

(18) $$\bar{R}\approx \log_{2}\left(1+E\left[\gamma \right]\right)-\frac{Q^{-1}\left(\varepsilon \right)}{\sqrt{L}\ln2},$$

where $E\left[\gamma \right]=\rho \left(\mu_{h}^{2}+\sigma_{h}^{2}\right)$. Our objective is to maximize the average MAR. Then, the antenna geometry optimization problem is formulated as

(19) $$\begin{array}{cc} P1:\; & \max_{X}\;\log_{2}\left(1+E\left[\gamma \right]\right)-\frac{Q^{-1}\left(\varepsilon \right)}{\sqrt{L}\ln2}\\ s.t.\; & C1:x_{n,m}-x_{n,m-1}\geq \Delta x,\;\forall n\in N,\;m=2,\ldots ,M,\\ \; & C2:0\leq x_{n,m}\leq L_{\max},\;\forall n,m. \end{array}$$

P1 is highly non-convex with respect to X due to the phase coupling in E[γ].

### 3.2. PPO-Based Geometry Optimization in the Phase Domain

To solve the non-convex problem P1, we propose a PPO-based framework that performs policy learning in the phase domain instead of the coordinate domain. The rationale is that the received signal quality is primarily determined by phase alignment, whereas direct position control induces a highly non-smooth action landscape under geometric constraints. Accordingly, the agent outputs phase domain actions, which are then mapped to feasible antenna locations via a PDAM. For notational consistency, the superscript *t* is used to denote time steps for scalar variables (e.g., $x_{n,m}^{\left(t\right)}$) and sets (e.g., $Q_{n,m}^{\left(t\right)}$), while the subscript *t* is used for vectors and matrices (e.g., $s_{t}$ and $X_{t}$). The actor is parameterized by a stochastic policy $\pi_{\theta }\left(a_{t}|s_{t}\right)$ with parameter vector θ, and the critic is parameterized by a value function $V_{\psi }\left(s_{t}\right)$ with parameter vector ψ. Both networks are implemented as multilayer perceptrons with two hidden layers of 256 neurons, each followed by ReLU activation and layer normalization. Specifically, the workflow of our proposed PPO-based algorithm for solving the optimization problem is presented in Algorithm 1.
**Algorithm 1** PPO With PDAM for PASS Geometry Optimization.**Require:** Initial actor parameters θ, initial critic parameters ψ, number of episodes $N_{\mathrm{ep}}$, and horizon *T***Ensure:** Optimized geometry $X^{★}$ and the corresponding maximum reward $r^{★}$  1: Initialize the actor policy $\pi_{\theta }$ and critic $V_{\psi }$.  2: Initialize the trajectory buffer D.  3: Set $X^{★}=0$ and $r^{★}=-\infty$.  4: **for** episode $e=1,2,\ldots ,N_{\mathrm{ep}}$ **do**  5:     Reset the environment and obtain the initial state $s_{0}$.  6:     **for** time step t=0,1,…,T−1 **do**  7:         Sample action $a_{t}∼\pi_{\theta }\left(\cdot |s_{t}\right)$.  8:         Recover the target phase vector from $a_{t}$.  9:         Map the target phase vector to a feasible geometry $X_{t}$ via PDAM.10:         Execute $X_{t}$, and observe reward $r^{\left(t\right)}$ and next state $s_{t+1}$.11:         Store $\left(s_{t},a_{t},r^{\left(t\right)},s_{t+1}\right)$ in D.12:         **if** $r^{\left(t\right)}>r^{★}$ **then**13:             $r^{★}\leftarrow r^{\left(t\right)}$, $X^{★}\leftarrow X_{t}$.14:         **end if**15:      **end for**16:      Estimate the advantages from D using generalized advantage estimation.17:      Update θ by maximizing the PPO clipped surrogate objective.18:      Update ψ by minimizing the value-function loss.19:      Clear the trajectory buffer D.20: **end for**21: **return** $X^{★}$ and $r^{★}$.

#### 3.2.1. State Space

Let $X_{t}\in R^{N\times M}$ denote the antenna geometry matrix at time step *t*, whose *n*-th row is given by

(20) $$x_{n,t}={\left[\begin{array}{c} x_{n,1}^{\left(t\right)},x_{n,2}^{\left(t\right)},\ldots ,x_{n,M}^{\left(t\right)} \end{array}\right]}^{T}\in R^{M}.$$

To improve numerical stability, the geometry is normalized by the waveguide length $L_{\max}$, yielding

(21) $${\bar{x}}_{t}=\frac{1}{L_{\max}}{\left[\begin{array}{c} x_{1,t}^{T},x_{2,t}^{T},\ldots ,x_{N,t}^{T} \end{array}\right]}^{T}\in R^{NM}.$$

The normalized user-location vector is defined as

(22) $$\bar{u}=\frac{p_{u}}{D_{\mathrm{ref}}}\in R^{3},$$

where $D_{\mathrm{ref}}$ is a reference distance. The state is then constructed as

(23) $$s_{t}={\left[\begin{array}{c} {\bar{x}}_{t}^{T},{\bar{u}}^{T},d_{wg} \end{array}\right]}^{T}\in R^{NM+4}.$$

#### 3.2.2. Action Space

Instead of directly outputting antenna positions, the actor generates a phase-control action for all NM antennas. Let

(24) $$\phi_{t}={\left[\begin{array}{c} \phi_{1}^{\left(t\right)},\phi_{2}^{\left(t\right)},\ldots ,\phi_{NM}^{\left(t\right)} \end{array}\right]}^{T}\in R^{NM}$$

denote the target phase vector. To avoid the discontinuity at the phase boundary, each phase is represented by its sine and cosine components. Accordingly, the action vector is defined as

(25) $$a_{t}=\left[\begin{array}{c} \sin(\phi_{t})\\ \cos(\phi_{t}) \end{array}\right]\in {\left[-1,1\right]}^{2NM},$$

where the sine and cosine functions are applied element-wise. Hence, the target phase associated with the *m*-th flattened antenna index can be recovered as

(26) $${\hat{\phi }}_{m}^{\left(t\right)}=\mathrm{atan}2\;\left(a_{m}^{\left(t\right)},a_{NM+m}^{\left(t\right)}\right),\;m=1,2,\ldots ,NM.$$

#### 3.2.3. PDAM

Since the PASS directly controls antenna positions rather than phases, the recovered target phase must be mapped to a feasible geometry. Let

(27) q=(n−1)M+m,n∈N,m∈M,

denote the flattened index corresponding to the *m*-th antenna on the *n*-th waveguide. For a candidate position $x\in [0,L_{\max}]$, the effective deterministic phase is modeled as

(28) $$\phi_{n,m}\left(x\right)=\mathrm{wrap}\;\left(-\beta_{g}x-\frac{2\pi }{\lambda }∥p_{n,m}\left(x\right)-p_{u}∥\right),$$

where wrap(·) is the phase-wrapping function that maps a real-valued phase to the principal interval (−π,π], defined as

(29) $$\mathrm{wrap}\left(z\right)=z-2\pi \left\lfloor \frac{z+\pi }{2\pi }\right\rfloor ,\;z\in R.$$

Since the phase is periodic modulo 2π, a given target phase may correspond to multiple candidate positions. We therefore first construct the phase-consistent candidate set

(30) $$Q_{n,m}^{\left(t\right)}=\left\{x\in \left[0,L_{\max}\right]\;|\;\left|\mathrm{wrap}\;\left(\phi_{n,m}\left(x\right)-{\hat{\phi }}_{q}^{\left(t\right)}\right)\right|\leq \Delta_{\phi }\right\},$$

where $\Delta_{\phi }\in \left(0,\pi \right]$ denotes the phase-matching tolerance. Among all candidates in $Q_{n,m}^{\left(t\right)}$, the selected position is given by

(31) $${\tilde{x}}_{n,m}^{\left(t\right)}=\arg\min_{x\in Q_{n,m}^{\left(t\right)}}∥p_{n,m}\left(x\right)-p_{u}∥.$$

If $Q_{n,m}^{\left(t\right)}=⌀$, we instead adopt the minimum phase-mismatch rule, i.e.,

(32) $${\tilde{x}}_{n,m}^{\left(t\right)}=\arg\min_{x\in [0,L_{\max}]}\left|\mathrm{wrap}\;\left(\phi_{n,m}\left(x\right)-{\hat{\phi }}_{q}^{\left(t\right)}\right)\right|.$$

After obtaining the preliminary positions ${\left\{{\tilde{x}}_{n,m}^{\left(t\right)}\right\}}_{m=1}^{M}$ on each waveguide, they are sorted in ascending order and sequentially adjusted to satisfy constraints C1 and C2. Specifically, each candidate position is first clipped to $\left[0,L_{\max}\right]$, after which a left-to-right spacing-repair step is applied to enforce the minimum inter-element distance. The resulting feasible geometry is denoted by $X_{t}$.

The above PDAM preserves the physical meaning of the phase-domain action while enforcing the PASS deployment constraints. Specifically, the phase-consistent candidate generation captures the phase-alignment objective, while distance-aware selection and spacing repair yield a feasible geometry with favorable large-scale channel gain.

#### 3.2.4. Reward Function

The objective of the proposed framework is to maximize the average MAR under finite-blocklength transmission. Accordingly, the immediate reward at time step *t* is defined as

(33) $$r^{\left(t\right)}=\log_{2}\left(1+E\left[\gamma^{\left(t\right)}\right]\right)-\frac{Q^{-1}\left(\varepsilon \right)}{\sqrt{L}\ln2},$$

where $\gamma^{\left(t\right)}$ is the instantaneous received SNR at time step *t* corresponding to $X_{t}$.

### 3.3. Computational Complexity Analysis

Let $L_{1}^{\left(a\right)}$ and $L_{2}^{\left(a\right)}$ denote the numbers of neurons in the first and second hidden layers of the actor network, respectively, and let $L_{1}^{\left(c\right)}$ and $L_{2}^{\left(c\right)}$ denote those of the critic network. Since the state dimension is NM+4 and the action dimension is 2NM, the forward-pass complexity of the actor network is $O(\left(NM+4\right)L_{1}^{\left(a\right)}+L_{1}^{\left(a\right)}L_{2}^{\left(a\right)}+2NM\;L_{2}^{\left(a\right)})$, where the three terms correspond to the input-to-hidden, hidden-to-hidden, and hidden-to-output layers, respectively. Similarly, the forward-pass complexity of the critic network is $O(\left(NM+4\right)L_{1}^{\left(c\right)}+L_{1}^{\left(c\right)}L_{2}^{\left(c\right)}+L_{2}^{\left(c\right)})$. Therefore, the overall per-step computational complexity during training is $O(\left(NM+4\right)L_{1}^{\left(a\right)}+L_{1}^{\left(a\right)}L_{2}^{\left(a\right)}+2NM\;L_{2}^{\left(a\right)}+\left(NM+4\right)L_{1}^{\left(c\right)}+L_{1}^{\left(c\right)}L_{2}^{\left(c\right)}+L_{2}^{\left(c\right)})$. When all hidden layers have the same width, i.e., $L_{1}^{\left(a\right)}=L_{2}^{\left(a\right)}=L_{1}^{\left(c\right)}=L_{2}^{\left(c\right)}=L$, the complexity reduces to $O(2L^{2}+\left(4NM+9\right)L)$.

### 3.4. Average BLER Analysis

For the given blocklength L and number of data bits *F* (i.e., the MAR at the UE is R=F/L), the average BLER is a crucial performance metric to evaluate the reliability of the PASS-aided SPC system. According to finite-blocklength information theory, the average BLER $\bar{\varepsilon }$ over fading channels can be evaluated by taking the expectation of the instantaneous BLER over the SNR distribution, namely,

(34) $$\bar{\varepsilon }=E\left[\varepsilon \right]\approx \int_{0}^{\infty }Q\left(\frac{C(x)-R}{\sqrt{V(x)/L}}\right)f_{\gamma }\left(x\right)dx,$$

where $C\left(x\right)=\log_{2}\left(1+x\right)$ is the Shannon capacity. Due to the complex form of the *Q*-function and the non-linear dispersion term V(x), deriving an exact closed-form expression is intractable. Similar to [16], we approximate the function $Q\left(\frac{C(x)-R}{\sqrt{V(x)/L}}\right)$ using a highly accurate piecewise function W(x)

(35) $$W\left(x\right)=\left\{\begin{array}{cc} 1, & 0<x\leq v_{1},\\ 1-a{\left(x-v_{1}\right)}^{2}, & v_{1}<x\leq v_{2},\\ \frac{1}{2}-\kappa \left(x-\tau \right), & v_{2}<x\leq v_{3},\\ b{\left(x-v_{4}\right)}^{2}, & v_{3}<x\leq v_{4},\\ 0, & x>v_{4}, \end{array}\right.$$

where $\kappa =\frac{\sqrt{L}}{\sqrt{2\pi (2^{2R}-1)}}$, $\tau =2^{R}-1$, $v_{1}=\tau -\frac{1}{\kappa },v_{2}=\tau -\frac{1}{4\kappa },v_{3}=\tau +\frac{1}{3\kappa },v_{4}=\tau +\frac{3}{2\kappa }$, $a=\frac{4}{9}\kappa^{2}$, and $b=\frac{6}{49}\kappa^{2}$.

To derive the probability density function (PDF) of γ, we first determine the probability distributions of $|h_{\mathrm{total}}{|}^{2}$. Since we have $h_{\mathrm{total}}∼\mathrm{CN}\left(\mu_{h},\sigma_{h}^{2}\right)$, $|h_{\mathrm{total}}|$ follows $\mathrm{Rice}\left(K_{h},\Omega_{h}\right)$, where $K_{h}=\frac{\mu_{h}^{2}}{\sigma_{h}^{2}}$ and $\Omega_{h}=\mu_{h}^{2}+\sigma_{h}^{2}$. Consequently, the PDF of $|h_{\mathrm{total}}{|}^{2}$ can be derived as [31]

(36) $$f_{|h_{\mathrm{total}}{|}^{2}}\left(x\right)=\frac{K_{h}+1}{\Omega_{h}}e^{-K_{h}-\frac{\left(K_{h}+1\right)}{\Omega_{h}}x}I_{0}\left(2\sqrt{\frac{K_{h}\left(K_{h}+1\right)x}{\Omega_{h}}}\right).$$

The presence of the special function $I_{0}\left(\cdot \right)$ makes it intractable to derive a closed-form expression for the average BLER. Fortunately, ${\left|h_{\mathrm{total}}\right|}^{2}$ can be approximated by a mixture Gamma distribution [37], as presented in Lemma 1.

**Lemma** **1.** *For a random variable $X∼\mathrm{Rice}\left(K_{X},\Omega_{X}\right)$, $X^{2}$ approximately follows a mixture Gamma distribution, i.e., $f_{X^{2}}\left(x\right)\approx \sum_{i=1}^{I}\frac{\omega_{i}x^{i-1}e^{-\frac{x}{\zeta }}}{\Gamma \left(i\right)\zeta^{i}}$, where I denotes the number of terms, $\zeta =\frac{\Omega_{X}}{1+K_{X}}$, $\omega_{i}=\alpha_{i}\Gamma \left(i\right)\zeta^{i}$ with $\sum_{i=1}^{I}\omega_{i}=1$, $\alpha_{i}=\frac{\theta_{i}}{\sum_{j=1}^{I}\theta_{j}\Gamma \left(j\right)\zeta^{j}}$, and $\theta_{i}=\frac{K_{X}^{i-1}}{e^{K_{X}}\Gamma {\left(i\right)}^{2}\zeta^{i}}$.*

**Proof.**  Please refer to [37].    □

Using Lemma 1, we can approximate the PDF of ${\left|h_{\mathrm{total}}\right|}^{2}$ as $f_{{\left|h_{\mathrm{total}}\right|}^{2}}\left(x\right)\approx \sum_{i=1}^{I}\frac{\omega_{i}x^{i-1}e^{-\frac{x}{\zeta }}}{\Gamma \left(i\right)\zeta^{i}}$, where $\zeta =\frac{\Omega_{h}}{1+K_{h}}$, $\omega_{i}=\alpha_{i}\Gamma \left(i\right)\zeta^{i}$ with $\sum_{i=1}^{I}\omega_{i}=1$, $\alpha_{i}=\frac{\theta_{i}}{\sum_{j=1}^{I}\theta_{j}\Gamma \left(j\right)\zeta^{j}}$, and $\theta_{i}=\frac{K_{h}^{i-1}}{e^{K_{h}}\Gamma {\left(i\right)}^{2}\zeta^{i}}$. Furthermore, the PDF of γ can be approximated as

(37) $$f_{\gamma }\left(x\right)\approx \sum_{i=1}^{I}\frac{\omega_{i}x^{i-1}e^{-\frac{x}{\rho \zeta }}}{\Gamma \left(i\right){\left(\rho \zeta \right)}^{i}}.$$

Finally, the expression for $\bar{\varepsilon }$ is given by Lemma 2.

**Lemma** **2.** *For the given blocklength L, number of data bits F, and related parameters of the PASS, the average BLER $\bar{\varepsilon }$ at the UE is derived as*

(38) $$\begin{array}{cc} \bar{\varepsilon } & \approx \sum_{i=1}^{I}\frac{\omega_{i}}{\Gamma (i)}\gamma \left(i,\frac{v_{1}}{\rho \zeta }\right)+\left(1-av_{1}^{2}\right)\sum_{i=1}^{I}\frac{\omega_{i}}{\Gamma (i)}\left(\gamma \left(i,\frac{v_{2}}{\rho \zeta }\right)-\gamma \left(i,\frac{v_{1}}{\rho \zeta }\right)\right)\\ \; & \;-a\sum_{i=1}^{I}\frac{\omega_{i}{\left(\rho \zeta \right)}^{2}}{\Gamma (i)}\left(\gamma \left(i+2,\frac{v_{2}}{\rho \zeta }\right)-\gamma \left(i+2,\frac{v_{1}}{\rho \zeta }\right)\right)\\ \; & \;+2av_{1}\sum_{i=1}^{I}\frac{\omega_{i}\rho \zeta }{\Gamma (i)}\left(\gamma \left(i+1,\frac{v_{2}}{\rho \zeta }\right)-\gamma \left(i+1,\frac{v_{1}}{\rho \zeta }\right)\right)\\ \; & \;+\left(\frac{1}{2}+\kappa \tau \right)\sum_{i=1}^{I}\frac{\omega_{i}}{\Gamma (i)}\left(\gamma \left(i,\frac{v_{3}}{\rho \zeta }\right)-\gamma \left(i,\frac{v_{2}}{\rho \zeta }\right)\right)\\ \; & \;-\kappa \sum_{i=1}^{I}\frac{\omega_{i}\rho \zeta }{\Gamma (i)}\left(\gamma \left(i+1,\frac{v_{3}}{\rho \zeta }\right)-\gamma \left(i+1,\frac{v_{2}}{\rho \zeta }\right)\right)\\ \; & \;+b\sum_{i=1}^{I}\frac{\omega_{i}{\left(\rho \zeta \right)}^{2}}{\Gamma (i)}\left(\gamma \left(i+2,\frac{v_{4}}{\rho \zeta }\right)-\gamma \left(i+2,\frac{v_{3}}{\rho \zeta }\right)\right)\\ \; & \;-2bv_{4}\sum_{i=1}^{I}\frac{\omega_{i}\rho \zeta }{\Gamma (i)}\left(\gamma \left(i+1,\frac{v_{4}}{\rho \zeta }\right)-\gamma \left(i+1,\frac{v_{3}}{\rho \zeta }\right)\right)\\ \; & \;+bv_{4}^{2}\sum_{i=1}^{I}\frac{\omega_{i}}{\Gamma (i)}\left(\gamma \left(i,\frac{v_{4}}{\rho \zeta }\right)-\gamma \left(i,\frac{v_{3}}{\rho \zeta }\right)\right). \end{array}$$

**Proof.**  Please see Appendix A.    □

### 3.5. Blocklength Optimization

In this subsection, for the given *F*, we analyze the minimum blocklength required to satisfy the UE’s reliability constraint. Specifically, the optimization problem is formulated as

(39) $$\begin{array}{cc} \; & L^{*}=\min_{L}L\\ s.t. & C3:\;\bar{\varepsilon }\leq {\bar{\varepsilon }}^{\mathrm{th}},\\ \; & C4:L_{\min}\leq L\leq L_{\max},\\ \; & C5:L\in Z^{+}, \end{array}$$

where ${\bar{\varepsilon }}^{\mathrm{th}}$ denotes the average BLER threshold at the UE, and $L_{\min}$ and $L_{\max}$ represent the minimum and maximum values of the allowed transmission blocklength, respectively.

Since the average BLER is a monotonically decreasing function of L [16], we can adopt a bisection search to determine $L^{*}$ as shown in Algorithm 2. The range of the search space is limited to $\left[L_{\min},L_{\max}\right]$, and L takes integer values in the optimization procedure. The computational complexity is Olog2Lmax−LminLmax−Lmin22 [38] (Section 4.4.1).
**Algorithm 2** Blocklength Optimization.**Input:  ***N*, *M*, *K*, $\beta_{n,m}$, $X^{★}$, w, ρ, *F*, ${\bar{\varepsilon }}^{\mathrm{th}}$, $L_{\min}$, $L_{\max}$.**Output:**  Determine $L^{*}$.  1: Set $L^{-}=L_{\min}$, $L^{+}=L_{\max}$.  2: Compute the average BLER ${\bar{\varepsilon }}^{-}$ corresponding to $L^{-}$.  3: Compute the average BLER ${\bar{\varepsilon }}^{+}$ corresponding to $L^{+}$.  4: **if** ${\bar{\varepsilon }}^{+}>{\bar{\varepsilon }}^{\mathrm{th}}$ **then**  5:    Set $L^{*}=-1$, where −1 represents no solution;  6: **else if** ${\bar{\varepsilon }}^{-}⩽{\bar{\varepsilon }}^{\mathrm{th}}$ **then**  7:    Set $L^{*}=L_{\min}$;  8: **else**  9:    **while** $L^{+}-L^{-}\neq 1$ **do**10:        Set $\hat{L}=\left\lceil \left(L^{-}+L^{+}\right)/2\right\rceil$;11:        Compute the average BLER $\hat{\varepsilon }$ corresponding to $\hat{L}$;12:        **if** $\hat{\varepsilon }⩽{\bar{\varepsilon }}^{\mathrm{th}}$ **then**13:           Set $L^{+}=\hat{L}$;14:        **else**15:           Set $L^{-}=\hat{L}$;16:        **end if**17:    **end while**18:    Set $L^{*}=L^{+}$;19: **end if**20: **Return** $L^{*}$.

## 4. Numerical Results

In this section, numerical results are provided to verify the effectiveness of the proposed scheme. The used PASS employs N=4 parallel waveguides, each with M∈{4,8} PAs. Similar to [12], we consider the factory automation application. Furthermore, identical to that used in [6], our system operates at a carrier frequency of $f_{c}=15$ GHz. The waveguide parameters are set to $n_{g}=1.5$, $L_{\max}=2$ m, H=2 m, $d_{\mathrm{wg}}=1.5$ m, Δx=0.02 m, and δ=0.05. The UE is located at $p_{u}=\left(5,1,0\right)$ m. The channel and transmission parameters are set to η=2.2, $\xi_{0}=10^{-3}$, K=10, $\sigma^{2}=-80$ dBm [6], $L_{\min}=100$, and $L_{\max}=1832$ [39]. In the proposed PPO-based network, both the actor and critic networks are configured with two hidden layers of 256 neurons (i.e., L=256). The training hyperparameters are set to a learning rate of $3\times 10^{-4}$, a discount factor of 0.99, and a generalized advantage estimation (GAE) factor of 0.95. The surrogate objective uses a clipping coefficient of 0.2, a value loss coefficient of 0.5, and an entropy coefficient of 0.02. The network parameters are updated using four optimization epochs per iteration with a mini-batch size of 64. We compare the proposed algorithm with the following benchmarks:

1. *PSO [24]*: The conventional PSO algorithm directly optimizes the antenna position vector. The swarm consists of 50 particles evolved over a maximum of 300 iterations, with an inertia weight of 0.7 and acceleration coefficients $c_{1}=c_{2}=1.5$.
2. *PPO [25]*: The standard on-policy PPO agent operates directly in the position domain without the proposed phase-domain action representation or the PDAM repair mechanism. The actor-critic network comprises two hidden layers of 256 neurons each. The agent is trained with a learning rate of $3\times 10^{-4}$, a discount factor of 0.97, a GAE factor of 0.95, a clipping ratio of 0.2, an entropy coefficient of 0.03, and a mini-batch size of 64 over 6 update epochs per rollout. Training runs for 600 episodes of 80 environment steps each.
3. *SAC [26]*: The convolutional neural network (CNN)-based SAC agent employs maximum-entropy regularization. The actor encodes the antenna configuration via two convolutional layers (1→32→64 channels) followed by a fully connected module of 128 hidden units, while the critic employs a two-layer perceptron of 256 hidden units. The temperature parameter is fixed at 0.001, with a learning rate of $10^{-3}$, a discount factor of 0.995, and a soft-update coefficient of 0.01. A replay buffer of capacity $2\times 10^{5}$ is used with mini-batch size 256. Training runs for 600 episodes of 80 environment steps each.
4. *TD3 [27]*: The TD3 agent employs delayed policy updates and target policy smoothing. The actor and both critic networks are two-layer perceptrons with 256 hidden neurons and layer normalization. The key hyperparameters include a learning rate of $3\times 10^{-4}$, a discount factor of 0.99, a soft-update coefficient of 0.005, a target policy noise of 0.2 (clipped at ±0.5), and a delayed actor update frequency of every two critic steps. A replay buffer of capacity $10^{5}$ is used with mini-batch size 256. Training runs for 600 episodes of 80 environment steps each.

In the following figures, these algorithms are labeled as “Baseline PSO”, “Baseline PPO”, “Baseline SAC”, and “Baseline TD3”, respectively. All simulations are conducted on a computer equipped with Python 3.10, PyTorch 2.1, and an NVIDIA RTX 3090 Ti GPU.

**Figure 2:**
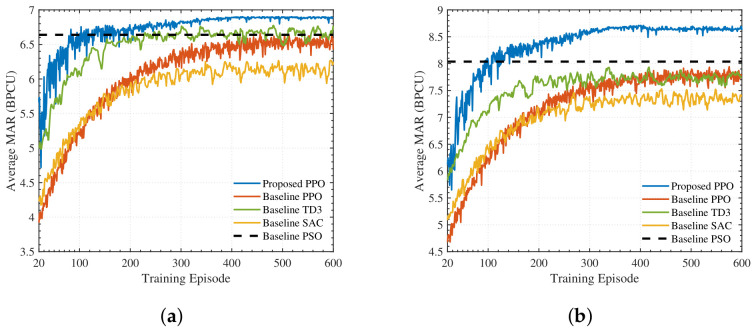
Reward versus training episode for (**a**) M=4 and (**b**) M=8.

Figure 2 plots the average MAR versus the training episodes for different numbers of PAs per waveguide under ρ=65 dB, L=200, and $\varepsilon =10^{-5}$. It is observed that all algorithms gradually converge to a stable performance as training proceeds for both M=4 and M=8, verifying the convergence and effectiveness of the learned placement policy. Furthermore, the proposed PPO-based algorithm converges after about 290 episodes for M=4 and 350 episodes for M=8 within the 600-episode training budget. Moreover, the converged performance of the proposed algorithm consistently outperforms that of all baseline algorithms, and the performance gain becomes more pronounced as *M* increases. Specifically, for M=8, the proposed algorithm achieves a clear gain over all baselines; for M=4, it also outperforms the baselines, albeit with a smaller gain. These results indicate that the proposed phase-aligned candidate generation and sequential decision mechanism can more effectively exploit the enlarged placement DoFs when more PAs are deployed on each waveguide, thereby yielding a higher average MAR. The detailed computational complexity metrics of the proposed algorithm are summarized in Table 2. Notably, the reported training time is measured on a single GPU. It can be substantially reduced by leveraging more powerful GPUs such as the RTX 4090 or by parallelizing the training across multiple GPUs. We observe from Table 2 that the peak GPU memory usage remains nearly constant at approximately 650 MB for both configurations, as the majority of the memory is consumed by the fixed PyTorch runtime and CUDA context overhead. The training time increases moderately from 5.1 min for M=4 to 6.9 min for M=8, as the forward-pass complexity scales roughly linearly with *M* while a fixed overhead dominates part of the runtime.

**Figure 3:**
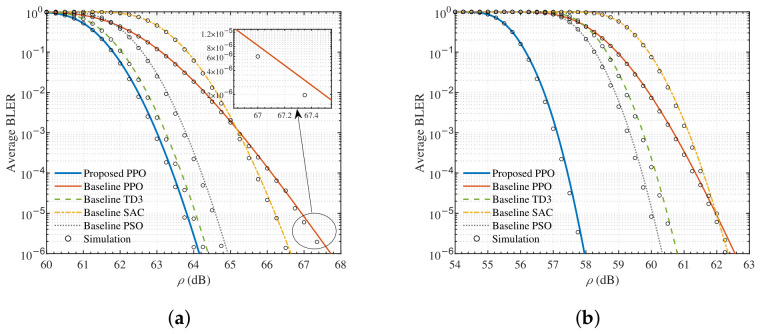
Average BLER versus transmit SNR for (**a**) M=4 and (**b**) M=8.

Figure 3 plots the average BLER versus the transmit SNR for all algorithms under L=200 and R=6 BPCU. It is observed that the simulation results closely match the analytical curves across the entire transmit SNR range. The maximum SNR gap between the analytical and simulated results (i.e., the difference in transmit SNR required to achieve the same average BLER) is less than 0.2 dB, which validates the accuracy of the derived average BLER analysis. Moreover, our proposed PPO-based algorithm consistently achieves a lower average BLER than all baselines at the same transmit SNR. The proposed algorithm exhibits a clear leftward shift of the average BLER curve compared with all baselines, indicating a noticeable power saving.

**Figure 4:**
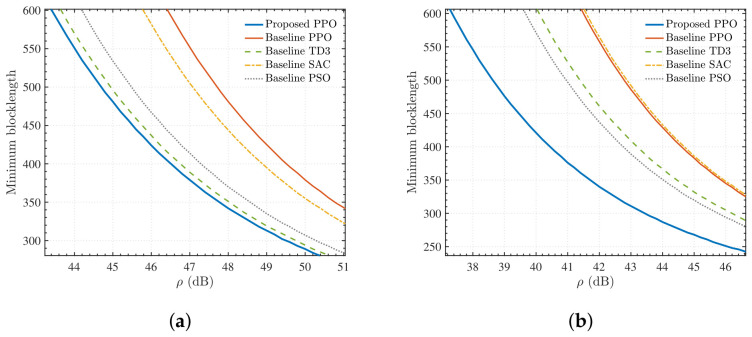
Minimum blocklength versus transmit SNR for (**a**) M=4 and (**b**) M=8.

Figure 4 plots the minimum blocklength versus the transmit SNR for all algorithms under ${\bar{\varepsilon }}^{\mathrm{th}}=10^{-5}$ and F=400. It is observed that, for all considered *M*, the proposed algorithm consistently requires a shorter blocklength than all baselines to achieve the same reliability target. For example, when M=4 and ρ=50 dB, the proposed PPO-based algorithm requires 289 channel uses, compared with 307 for Baseline PSO, 379 for Baseline PPO, 355 for Baseline SAC, and 294 for Baseline TD3, corresponding to reductions of 18, 90, 66, and 5 channel uses, respectively. This result indicates that the proposed sequential placement policy can more effectively exploit the spatial DoFs of PASSs, thereby significantly improving the finite-blocklength transmission efficiency.

## 5. Conclusions

This paper proposed a phase-domain DRL framework for geometry optimization in PASSs under SPC constraints. Unlike conventional coordinate-domain approaches, the proposed method operates in a dual-component trigonometric phase domain, thereby avoiding the phase discontinuity issue caused by the periodic nature of electromagnetic waves. The learned phase-domain actions are mapped to feasible antenna positions via a customized PDAM, which ensures physical collision avoidance while preserving the phase-alignment objective. To evaluate the system reliability, we derived an analytical expression for the average BLER based on a mixture Gamma approximation of the received SNR distribution. Then, a bisection search algorithm was developed to minimize the required blocklength under the user’s average BLER constraint. Simulation results validated the proposed theoretical analysis and demonstrated that the phase-domain PPO scheme outperforms the conventional baselines in terms of average MAR, average BLER, and blocklength efficiency, especially as the number of antennas per waveguide increases. These results confirm that the proposed learning-based framework offers a robust and efficient solution for PASS-aided URLLC systems. Practical implementation issues such as synchronization accuracy, hardware complexity, imperfect CSI, and user localization errors are left for future investigation. Moreover, experimental validation of the proposed system on a hardware prototype is also an important direction for future work.

## Figures and Tables

**Table 1:** Description of key symbols.

Symbol	Description
E[·]	Expectation
${\left(\cdot \right)}^{T}$	Transpose
${\left(\cdot \right)}^{H}$	Conjugate transpose
${\left(\cdot \right)}^{*}$	Conjugate
C	The complex domain
R	The real domain
$Z^{+}$	The set of positive integers
$\mathrm{CN}(\mu ,\sigma^{2})$	A complex Gaussian distribution with mean μ and variance $\sigma^{2}$
|·|	Absolute value of a real scalar or magnitude of a complex number
∥·∥	Euclidean norm
$I_{N}$	N×N identity matrix
$\mathrm{Rice}\left(K,\Omega \right)$	Rician distribution with Rician factor *K* and average power Ω
Γ(·)	Gamma function [31]
γ(·,·)	Lower incomplete Gamma function [31]
Q(·)	Gaussian *Q*-function [31]
$\mathrm{sinc}\left(x\right)=\frac{\sin(\pi x)}{\pi x}$	Sinc function
diag(·)	Forms a diagonal matrix from inputs
⌈·⌉	Ceiling function
⌊·⌋	Floor function
$I_{0}\left(\cdot \right)$	The zero-order modified Bessel function of the first kind
${\left[X\right]}_{i,j}$	The (i,j)-th entry of matrix X
atan2(y,x)	Four-quadrant inverse tangent in (−π,π]
O(·)	Order of computational complexity

**Table 2:** Computational complexity metrics of the proposed algorithm.

*M*	Peak GPU Memory	Training Time (min)	Convergence Episodes
4	652 MB	5.1	≈290
8	657 MB	6.9	≈350

## Data Availability

The data presented in this study are available on request from the corresponding author.

## Appendix A. Proof of Lemma 2

Given that $f_{\gamma }\left(x\right)\approx \sum_{i=1}^{I}\frac{\omega_{i}x^{i-1}e^{-\frac{x}{\rho \zeta }}}{\Gamma \left(i\right){\left(\rho \zeta \right)}^{i}}$, we obtain

(A1) $$\begin{array}{cc} \bar{\varepsilon } & \approx \int_{0}^{\infty }Q\left(\frac{C(x)-R}{\sqrt{V(x)/L}}\right)f_{\gamma }\left(x\right)dx\\ \; & =\underset{I_{1}}{\underset{︸}{\int_{0}^{v_{1}}f_{\gamma }\left(x\right)dx}}+\underset{I_{2}}{\underset{︸}{\int_{v_{1}}^{v_{2}}\left(1-a{\left(x-v_{1}\right)}^{2}\right)f_{\gamma }\left(x\right)dx}}\\ \; & \;+\underset{I_{3}}{\underset{︸}{\int_{v_{2}}^{v_{3}}\left(\frac{1}{2}-\kappa \left(x-\tau \right)\right)f_{\gamma }\left(x\right)dx}}+\underset{I_{4}}{\underset{︸}{\int_{v_{3}}^{v_{4}}b{\left(x-v_{4}\right)}^{2}f_{\gamma }\left(x\right)dx}} \end{array}$$

by using (35). First, $I_{1}$ can be written as

(A2) $$\begin{array}{c} I_{1}\approx \int_{0}^{v_{1}}\sum_{i=1}^{I}\frac{\omega_{i}x^{i-1}e^{-\frac{x}{\rho \zeta }}}{\Gamma \left(i\right){\left(\rho \zeta \right)}^{i}}dx=\sum_{i=1}^{I}\frac{\omega_{i}}{\Gamma \left(i\right){\left(\rho \zeta \right)}^{i}}\int_{0}^{v_{1}}x^{i-1}e^{-\frac{x}{\rho \zeta }}dx\overset{\left(a\right)}{=}\sum_{i=1}^{I}\frac{\omega_{i}}{\Gamma (i)}\gamma \left(i,\frac{v_{1}}{\rho \zeta }\right), \end{array}$$

where step (a) is obtained by using [31] (Equation 3.381). Second, $I_{2}$ can be written as

(A3) $$\begin{array}{cc} I_{2} & \approx \int_{v_{1}}^{v_{2}}\left(1-a{\left(x-v_{1}\right)}^{2}\right)\sum_{i=1}^{I}\frac{\omega_{i}x^{i-1}e^{-\frac{x}{\rho \zeta }}}{\Gamma \left(i\right){\left(\rho \zeta \right)}^{i}}dx\\ \; & =\left(1-av_{1}^{2}\right)\sum_{i=1}^{I}\underset{I_{2,1}}{\underset{︸}{\int_{v_{1}}^{v_{2}}\frac{\omega_{i}x^{i-1}e^{-\frac{x}{\rho \zeta }}}{\Gamma \left(i\right){\left(\rho \zeta \right)}^{i}}dx}}-a\sum_{i=1}^{I}\underset{I_{2,2}}{\underset{︸}{\int_{v_{1}}^{v_{2}}\frac{\omega_{i}x^{i+1}e^{-\frac{x}{\rho \zeta }}}{\Gamma \left(i\right){\left(\rho \zeta \right)}^{i}}dx}}\\ \; & \;+2av_{1}\sum_{i=1}^{I}\underset{I_{2,3}}{\underset{︸}{\int_{v_{1}}^{v_{2}}\frac{\omega_{i}x^{i}e^{-\frac{x}{\rho \zeta }}}{\Gamma \left(i\right){\left(\rho \zeta \right)}^{i}}dx}}, \end{array}$$

where $I_{2,1}$, $I_{2,2}$, and $I_{2,3}$ are derived as

(A4) $$\begin{array}{cc} I_{2,1} & =\frac{\omega_{i}}{\Gamma \left(i\right){\left(\rho \zeta \right)}^{i}}\int_{v_{1}}^{v_{2}}x^{i-1}e^{-\frac{x}{\rho \zeta }}dx\\ \; & =\frac{\omega_{i}}{\Gamma \left(i\right){\left(\rho \zeta \right)}^{i}}\left(\int_{0}^{v_{2}}x^{i-1}e^{-\frac{x}{\rho \zeta }}dx-\int_{0}^{v_{1}}x^{i-1}e^{-\frac{x}{\rho \zeta }}dx\right), \end{array}$$

(A5) $$\begin{array}{cc} I_{2,2} & =\frac{\omega_{i}}{\Gamma \left(i\right){\left(\rho \zeta \right)}^{i}}\int_{v_{1}}^{v_{2}}x^{i+1}e^{-\frac{x}{\rho \zeta }}dx\\ \; & =\frac{\omega_{i}}{\Gamma \left(i\right){\left(\rho \zeta \right)}^{i}}\left(\int_{0}^{v_{2}}x^{i+1}e^{-\frac{x}{\rho \zeta }}dx-\int_{0}^{v_{1}}x^{i+1}e^{-\frac{x}{\rho \zeta }}dx\right), \end{array}$$

and

(A6) $$\begin{array}{cc} I_{2,3} & =\frac{\omega_{i}}{\Gamma \left(i\right){\left(\rho \zeta \right)}^{i}}\int_{v_{1}}^{v_{2}}x^{i}e^{-\frac{x}{\rho \zeta }}dx\\ \; & =\frac{\omega_{i}}{\Gamma \left(i\right){\left(\rho \zeta \right)}^{i}}\left(\int_{0}^{v_{2}}x^{i}e^{-\frac{x}{\rho \zeta }}dx-\int_{0}^{v_{1}}x^{i}e^{-\frac{x}{\rho \zeta }}dx\right), \end{array}$$

respectively. Then, applying [31] (Equation 3.381) again, we obtain $I_{2,1}=\frac{\omega_{i}}{\Gamma (i)}\left(\gamma \left(i,\frac{v_{2}}{\rho \zeta }\right)-\gamma \left(i,\frac{v_{1}}{\rho \zeta }\right)\right)$, $I_{2,2}=\frac{\omega_{i}{\left(\rho \zeta \right)}^{2}}{\Gamma (i)}\left(\gamma \left(i+2,\frac{v_{2}}{\rho \zeta }\right)-\gamma \left(i+2,\frac{v_{1}}{\rho \zeta }\right)\right)$, and $I_{2,3}=\frac{\omega_{i}\rho \zeta }{\Gamma (i)}\left(\gamma \left(i+1,\frac{v_{2}}{\rho \zeta }\right)-\gamma \left(i+1,\frac{v_{1}}{\rho \zeta }\right)\right)$. Finally, $I_{3}$ and $I_{4}$ can be respectively derived as

(A7) $$\begin{array}{cc} I_{3} & \approx \int_{v_{2}}^{v_{3}}\left(\frac{1}{2}-\kappa \left(x-\tau \right)\right)\sum_{i=1}^{I}\frac{\omega_{i}x^{i-1}e^{-\frac{x}{\rho \zeta }}}{\Gamma \left(i\right){\left(\rho \zeta \right)}^{i}}dx\\ \; & =\int_{v_{2}}^{v_{3}}\sum_{i=1}^{I}\frac{\omega_{i}x^{i-1}e^{-\frac{x}{\rho \zeta }}}{\Gamma \left(i\right){\left(\rho \zeta \right)}^{i}}dx-\kappa \int_{v_{2}}^{v_{3}}\sum_{i=1}^{I}\frac{\omega_{i}x^{i}e^{-\frac{x}{\rho \zeta }}}{\Gamma \left(i\right){\left(\rho \zeta \right)}^{i}}dx\\ \; & =\left(\frac{1}{2}+\kappa \tau \right)\sum_{i=1}^{I}\frac{\omega_{i}}{\Gamma (i)}\left(\gamma \left(i,\frac{v_{3}}{\rho \zeta }\right)-\gamma \left(i,\frac{v_{2}}{\rho \zeta }\right)\right)\\ \; & \;-\kappa \sum_{i=1}^{I}\frac{\omega_{i}\rho \zeta }{\Gamma (i)}\left(\gamma \left(i+1,\frac{v_{3}}{\rho \zeta }\right)-\gamma \left(i+1,\frac{v_{2}}{\rho \zeta }\right)\right), \end{array}$$

and

(A8) $$\begin{array}{cc} I_{4} & \approx \int_{v_{3}}^{v_{4}}b{\left(x-v_{4}\right)}^{2}\sum_{i=1}^{I}\frac{\omega_{i}x^{i-1}e^{-\frac{x}{\rho \zeta }}}{\Gamma \left(i\right){\left(\rho \zeta \right)}^{i}}dx\\ \; & =b\int_{v_{3}}^{v_{4}}\sum_{i=1}^{I}\frac{\omega_{i}x^{i+1}e^{-\frac{x}{\rho \zeta }}}{\Gamma \left(i\right){\left(\rho \zeta \right)}^{i}}dx-2bv_{4}\int_{v_{3}}^{v_{4}}\sum_{i=1}^{I}\frac{\omega_{i}x^{i}e^{-\frac{x}{\rho \zeta }}}{\Gamma \left(i\right){\left(\rho \zeta \right)}^{i}}dx+bv_{4}^{2}\int_{v_{3}}^{v_{4}}\sum_{i=1}^{I}\frac{\omega_{i}x^{i-1}e^{-\frac{x}{\rho \zeta }}}{\Gamma \left(i\right){\left(\rho \zeta \right)}^{i}}dx\\ \; & =b\sum_{i=1}^{I}\frac{\omega_{i}{\left(\rho \zeta \right)}^{2}}{\Gamma (i)}\left(\gamma \left(i+2,\frac{v_{4}}{\rho \zeta }\right)-\gamma \left(i+2,\frac{v_{3}}{\rho \zeta }\right)\right)\\ \; & \;-2bv_{4}\sum_{i=1}^{I}\frac{\omega_{i}\rho \zeta }{\Gamma (i)}\left(\gamma \left(i+1,\frac{v_{4}}{\rho \zeta }\right)-\gamma \left(i+1,\frac{v_{3}}{\rho \zeta }\right)\right)\\ \; & \;+bv_{4}^{2}\sum_{i=1}^{I}\frac{\omega_{i}}{\Gamma (i)}\left(\gamma \left(i,\frac{v_{4}}{\rho \zeta }\right)-\gamma \left(i,\frac{v_{3}}{\rho \zeta }\right)\right). \end{array}$$
